# DNA Scaffolded Silver Clusters: A Critical Study

**DOI:** 10.3390/molecules21020216

**Published:** 2016-02-17

**Authors:** Bidisha Sengupta, Christa Corley, Keith Cobb, Anthony Saracino, Steffen Jockusch

**Affiliations:** 1 Department of Chemistry, Tougaloo College, 500 West County Line Road, Tougaloo, MS 39174, USA; christa.lanay@gmail.com (C.C.); kcobbjr.21@gmail.com (K.C.); tonysaracino@hotmail.com (A.S.); 2 Department of Chemistry, Columbia University, 3000 Broadway, New York, NY 10027, USA; sj67@columbia.edu

**Keywords:** i-motif, microenvironment, circular dichroism, fluorescence, size exclusion chromatography

## Abstract

Fluorescent silver nanoclusters (Ag-NCs) are in prominence as novel sensing materials due to their biocompatibility, photostability, and molecule-like optical properties. The present work is carried out on an array (17 sequences) of 16 bases long cytosine rich, single stranded DNA templates 5′-C_3_X_i_C_3_X_ii_C_3_X_iii_C_3_X_iv_-3′ where i, ii, iii, iv correspond to T/G/C deoxynucleobases (with default base A). Among all the oligonucleotides, a sequence C_3_AC_3_AC_3_TC_3_G (3T4G) has been identified, which grows three different near-infrared-emitting NC species with absorption/emission maxima at ~620/700 (species I), 730/800 (species II), and 830 (Species III) nm, respectively. The nature of the spectral profiles, along with relevant parameters namely absorption maximum ($\lambda_{\text{abs}}^{\text{max}}$), emission maximum ($\lambda_{\text{em}}^{\text{max}}$), anisotropy (*r*), lifetime (τ), circular dichroism spectral data are used to understand the microenvironments of the fluorescent NC species I, II, and III. DNA:Ag stiochiometric, pH and solvent dependent studies proved that i-motif scaffolds with different folding topologies are associated with the growth of these three species and a certain concentration of silver and H^+^ favor the growth of species III. Size exclusion chromatographic measurements provided similar indications that a folded, more compact, classic i-motif template is associated with the formation of the longer NIR (~830 nm) absorbing species. This study provides a more definitive approach to design and obtain a targeted DNA templated Ag-NC with required emission properties for biophysical and cellular applications.

## 1. Introduction

Metal nanoclusters (NCs) with sizes around 1 nm or less, have discrete electronic transitions, which give rise to light absorption and emission [1,2,3,4,5]. These metallic NCs with no apparent plasmonic characteristics, draw the link between metal atoms and nanoparticles and exhibit molecule-like properties [4,5]. Fluorescent, biocompatible (water-soluble) silver (Ag) and gold (Au)-NCs can be prepared by using template-based synthetic strategies [3,4].

Compared to organic dyes and quantum dots, DNA scaffolded Ag-NCs are more photostable and less prone to blinking on long timescales [6,7,8]. The NCs act as good fluorescent probes in fields like optical sensing [9], detection of trace metals [10,11], small biomolecules [12,13], protein [14], enzymes [15], and single nucleotide modification in DNA [8]. Characterized by excellent brightness and photostability, the optical properties of the enclosed silver clusters not only vary with (host:silver) stoichiometry, oxidation number, and geometry, but also by the interactions with their encapsulating matrix [16,17]. However, small variations in the synthetic procedure may lead to the formation of large non-fluorescent plasmonic silver nanoparticles [16].

DNA sequences and secondary structures play a critical role in dictating the fluorescence emission properties of the DNA enclosed Ag-NCs from the blue to near-infrared region with variable quantum yields up to 64% [6,16,18]. Among the four bases, the proximity of guanine (G) is found to play an important role in the enhancement of the DNA templated Ag-NC fluorescence (to more than 500-fold) in the red region, which has been used in the design of Nano Cluster Beacons [6]. Single-molecule imaging and fluorescence correlation spectroscopic results depicted that the increase in fluorescence was found to grow exponentially with an increasing number of guanine (G) bases, when brought into proximity of Ag clusters. Charge transfer-based fluorescence can be the reason for the observed on/off in guanine-proximity-induced emission [6]. It has been observed that cytosine (C) interacts with Ag^+^ strongly, while adenine (A) and thymine (T) have weaker influence [7] in the formation of Ag-NC.

Although some studies have been conducted correlating DNA sequences and lengths to the encapsulated Ag-NC [19], the significance of the secondary structures of the DNA templates on the emission properties of the enclosed AG-NCs, was not discussed. Hence further studies are necessary to understand the role of the microenvironments on the luminescence properties of the Ag-NCs. Near infrared (NIR) emissive and highly stable bio-probes have great potential in bioimaging, (where absorption and scattering from endogenous substances are minimized) [17,20], and hence the focus of this study is on NIR emissive Ag-NCs with emission ≥700 nm. Here we provided a systematic study on the spectroscopic properties of Ag-NCs on an array of single stranded DNA oligonucleotide templates, whose sequences differ in one or two bases.

With the common base template as (5′-C_3_X_i_C_3_X_ii_C_3_X_iii_C_3_X_iv_-3′) where X_i_, X_ii_, X_iiii_, X_iv_ are C/T/G with various combinations producing 17 single-stranded sequences (with default X to be A), Ag-NCs were synthesized in 10 mM citrate buffer of pH 6.5. We isolated a sequence 5′-C_3_AC_3_AC_3_TC_3_G-3′ which grows three different kind of species emitting at ≥700 nm: Species I (λ_ex_ = 620/λ_em_ = 700), Species II (λ_ex_ = 730/λ_em_ = 800), Species III (λ_abs_ = 830). Circular dichroism spectroscopic measurements at different pH and solvents dictate the importance of the secondary structure in the formation of the NIR emitting species. Solvents of different polarities and a confined matrix namely methyl-beta-cyclodextrin (CDx) were used to understand the solvent dipolar relaxation and stabilities of the three species. With time, species III slowly converts to species II and I which can be used to tune the formation of a species over the other. Size exclusion chromatographic studies implied the compactness (folded template) of the three species to be III > II > I. It is likely that by isolating and understanding the properties of the stable NIR emissive DNA enclosed Ag-NCs, programmed synthesis of DNA-Ag NCs as NIR imaging probes can be possible [21].

## 2. Results and Discussions

### 2.1. Studies on an Array of Single Stranded DNA Templates

**Figure 1:**
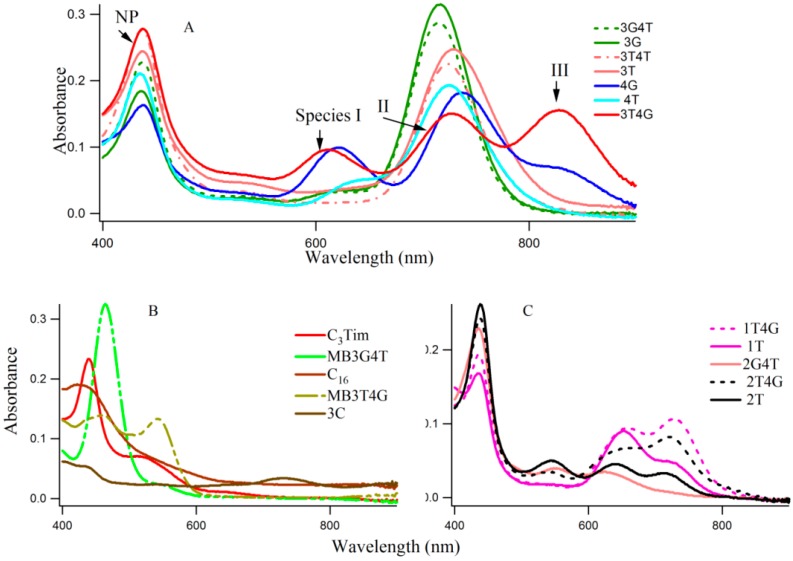
(**A**–**C**) show typical absorption spectra of silver nanolcusters on various DNA templates (15 µM DNA with 90 μM AgNO_3_ and 90 µM NaBH_4_). The sequences of the DNA templates are given in Scheme 1.

**Scheme 1:**
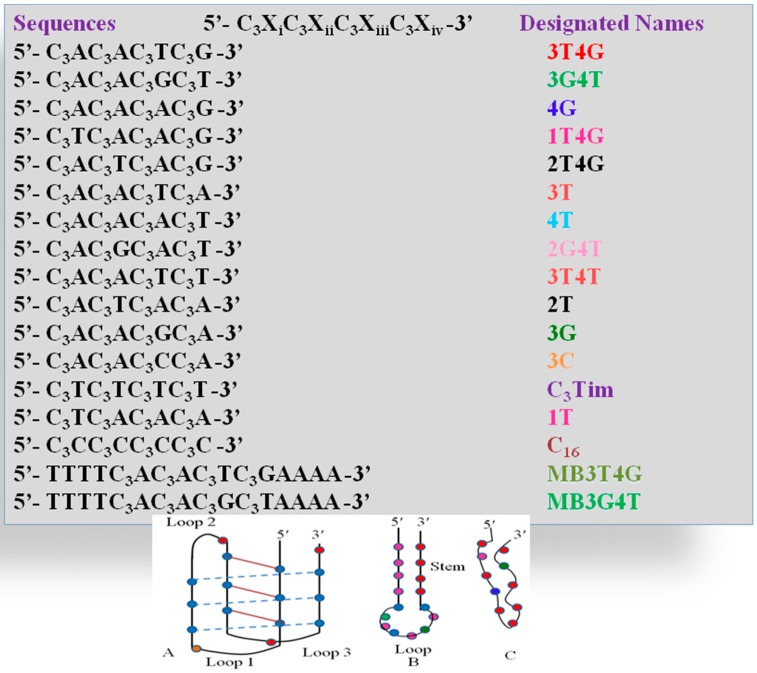
(**Top**): Sequences of all the single stranded DNA templates and their corresponding label names, which are used in the study; (**Bottom**): Unusual motifs which the DNA templates adapt during the Ag-NC formation; (**A**) C_4_imotif; (**B**) Hairpin loop; (**C**) Random fold.

Figure 1A–C shows typical absorption spectra of Ag-NCs on seventeen oligonucleotide templates (see Scheme 1 for sequences of oligonucleotides). The absorption of the nucleobases in the UV region are not shown here. The Ag-NCs are made in pH 6.5 citrate buffer with DNA (15 μM), AgNO_3_ (90 μM) and NaBH_4_ (90 μM) following the protocol as discussed below. Silver nanoparticles (NP) are formed during the process, which is evident by the absorption band ~400–420 nm [3]. A [DNA]:[Ag] of 1:6, was chosen to optimize the Ag nanoparticle formation, while forming all the fluorescing species of the Ag-NC on a particular DNA template. Examination of the absorption spectra in Figure 1 reveals three distinct spectral regions which were assigned to three different species of Ag-NC: species I (620–650 nm), species II (720–750 nm), and species III (~800). The population of these three species appear to be strongly dependent on the sequence of the oligonucleotides (see also Table 1). The presence of “G” at X_iv_ with “T” at the X_iii_ positions seem to play important roles in the formation of the three species (e.g., 3T4G), while the reverse is not true (e.g., 3G4T, forms mainly species II similar to 3G, 3T, and 3T4T). The template 4G forms species I and II with a partial III. Other sequences with G at X_iv_, while “T” at X_i_ or X_ii_ does not form similar NCs. When “T” or “C” is present at all X_s_, the absorption spectra are drastically different. The template 3G forms Ag-NCs that are different from 4G. When a stretch of four complementary bases (T_4_ and A_4_) is added to the 5′ and 3′ ends of 3T4G and 3G4T to form MB3T4G (greenish-yellow solid line) and MB3G4T (light green line) in Figure 1, the absorption spectra change drastically. It is evident from the sequences of MB3T4G and MB3G4T that the addition of the T_4_ and A_4_ stretches help the DNA to fold like a hairpin (Scheme 1B), which is responsible for the dramatic differences between the absorption spectra of 3T4G and MB3T4G as well as 3G4T and MB3G4T. These results suggest that although the secondary interactions of the template are destroyed by heating at boiling temperature during the Ag-NC synthesis (see the Experimental Section below), the Ag-NC conjugated scaffold renatures back depending on its intrinsic sequence. This is further discussed below in the CD section. The size of the nanocluster and the sites of its formation dictate its optical properties.

**Figure 2:**
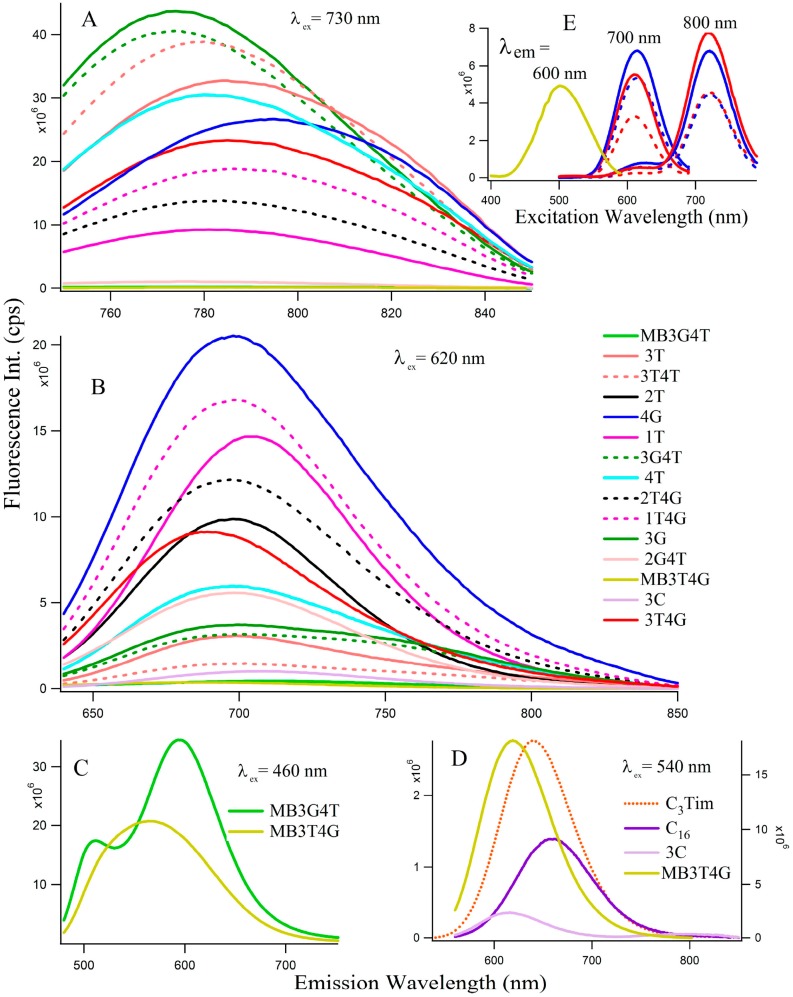
Fluorescence emission spectra of the silvers nanoclusters on various DNA templates (15 µM DNA with 90 μM AgNO_3_ and 90 µM NaBH_4_). The excitation wavelengths (λ_ex_) are stated in each figure (**A**–**D**). Figure (**E**) represents excitation spectra at the emission wavelengths 600 nm (for MB3T4G), 700 and 800 nm (for 4G-**blue** and 3T4G-**red** in buffer, solid lines and in methyl-beta-cyclodextrin, dashed lines).

Figure 2 depicts the fluorescence spectra of the Ag-NCs grown on the seventeen oligonucleotide templates. Depending on the absorption profiles, the NCs are excited at 730 nm (Figure 2A, species II) and 620 nm (Figure 2B, species I) for most of the NCs, except the ones on C_16_, C_3_Tim, MB3T4G, and MB3G4T templates where λ_ex_ = 460 nm (Figure 2C) and 540 nm (Figure 2D) nm. Table 1 summarizes the fluorescence maxima ($\lambda_{\text{em}}^{\text{max}}$) of all the species for the 17 templates. The Figure 2E represents fluorescence excitation spectra monitored at emission wavelengths of 600 nm for MB3T4G (yellow), 700 nm and 800 nm for both 4G (blue) and 3T4G (red) in buffer (solid lines) and in CDx (dashed lines) environments, where observed $\lambda_{\text{em}}^{\text{max}}$ are ~500 nm for MB3T4G and 610, 720 nm for both 4G (blue) and 3T4G (red) templates. The observation of a small excitation band at ~610 nm for emission wavelength at 800 nm indicates the possibility of FRET [22,23] between species I and II. It might be happening if two clusters are forming on the same template within a few nanometers, however further studies are needed to confirm.

Figure 1 and Figure 2 reveal that the oligonucleotides (5′-C_3_X_i_C_3_X_ii_C_3_X_iii_C_3_X_iv_-3′) with “G” and “T” at X_iii_ and X_iv_ are important to grow species I and II which emit at ~700 and 800 nm, respectively. 3T4G and MB3T4G have drastically different fluorescence emission spectra, which supports observations from Figure 1. These observations raised the importance of studying the secondary structures of these single stranded DNA oligonucleotides. Due to the limitation of the spectral range of the detector in the spectrofluorometer used for this study, no steady state emission studies with 830 nm excitation were performed. However, the size exclusion chromatographic measurements using the fluorescence detector allowed us to study the species III at 830 nm/860 nm (λ_ex_/λ_em_), which is discussed below.

Preliminary fluorescence lifetime measurements were performed with the aim of obtaining better insights regarding the distributions and microenvironments of the Ag-NCs on the C-rich DNA templates [24]. Table 2 lists the average fluorescence lifetimes ($\bar{\tau }$) along with double exponential decay components (τ_1_, τ_2_ ns) for species I of selected DNA templates and Figure S1 presents the decay profiles. Although there is no difference in ($\bar{\tau }$) between 3T4G, 3T and 4G in buffer, the population distribution of the minor (τ_1_) and major (τ_2_) components vary between 3T4G and 3T/4G scaffolds. The lifetime of the major component decreased from 2.8 (in 3T/4G ) to 2.3 ns in 3T4G which indicates that the microenvironments of the Ag-NC species I are different in the above matrices. There is a drastic difference of ($\bar{\tau }$) between Ag-NCs enclosed in 3T4G/4G/3T and MB3T4G, C_16_ and C_3_Tim templates, implying variations in microenvironments. This is discussed using CD spectroscopic measurements which are given below. The presence of two absorption bands in Figure 1 for MB3T4G templated Ag-NC indicated two silver nanocluster species which are supported by two very distinct ($\bar{\tau }$) of 1.7 and 4.6 ns with λ_ex_/λ_em_ = 460/535 and 496/620 nm, respectively. Two distinct lifetime components (biexponential decay), for mostly all decay studies (λ_ex_/λ_em_) on most of the nanoclusters, are indicative of heterogeneity in the microenvironment, [24,25] which is related to the various structural motifs (loops, grooves etc.) present on a DNA template (see Scheme 1A,B). It is pertinent to mention that unlike fluorescence emission ($\lambda_{\text{em}}^{\text{max}}$) which is usually environment sensitive, the observed lifetime value depends on both radiative as well as non-radiative decay parameters Equation (2) [24], some of which are likely to depend on other factors besides polarity or hydrophobicity of the surrounding.

**Figure 3:**
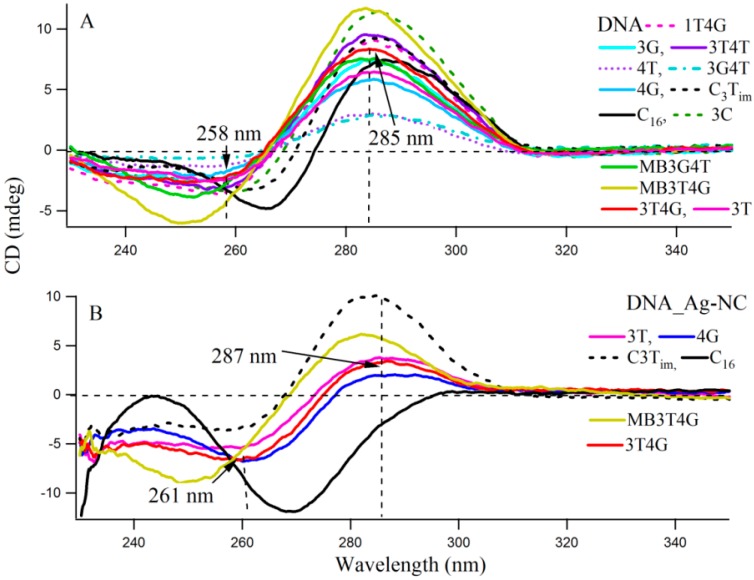
Circular Dichroism spectra of (**A**) 10 µM DNA and (**B**) 15 µM Ag-NC conjugated DNA at pH 6.5 citrate buffer solution. The vertical dashed lines show the wavelengths which characterize the formation of the unusual DNA structural motif, namely C_4_i-motif.

Circular dichroism (CD) spectra provide diagnostic signatures for the presence of secondary structures in the single stranded DNA sequence. A characteristic positive peak at ~288 nm and a negative peak at ~265 nm, with crossover at 276 nm, indicate the formation of an i-motif structure [26,27]. A major difference between the CD spectra of the spontaneously renatured naked DNA and Ag-NC conjugated DNA template is observable in Figure S2, which clearly indicates the importance of Ag-NC in the refolding process. Figure 3 presents the CD spectra of the DNA templates in free (A) and with Ag-NCs (B) at room temperature in 10 mM citrate buffer at pH 6.5. Most of the DNA sequences (see Scheme 1A) showed presence of weak i-motifs (with negative and positive bands at ~258 and 285 nm) except oligonucleotides C_16_, MB3T4G and MB3G4T which have distinct CD spectra. C_16_ shows positive and negative bands at 266 and 288 nm, while MB3T4G and MB3G4T show negative bands at ~252 nm. The CD spectra of the Ag-NC conjugated DNA templates showed characteristic negative and positive bands at ~261 and 287 nm (in Figure 3B) which indicates a classic i-motif folding of the scaffold. As is mentioned before, the DNA templates were denatured during the formation of the Ag-NCs. Hence the stronger features of i-motif (compared to Figure 3A) in the CD spectra of the Ag-NC conjugated DNA templates at pH 6.5, are indicative of Ag induced refolding of the templates. The presence of complementary bases at the two ends of MB3T4G and MB3G4T helps in folding these oligonucleotides in a unique hairpin form (Scheme 1B), which is responsible for the observed differences in their CD spectra compared to other templates in Figure 3. MB3T4G has five base pairs in the hairpin stem, while MB3G4T has four, which gives rise to the distinct microenvironments of the enclosed Ag-NC on MB3T4G/MB3G4T templates, and explains the dissimilarities between themselves. According to Dettler *et al.* [26], there are at least three i-motif-like folded conformations for a single cytosine rich DNA oligonucleotide, which can co-exist in a given solution. The observed CD spectra are an ensemble of all the i-motif-like conformers at a given condition and a single base change can give rise to a different i-motif. Hence the variations in the CD spectral profiles between all the C-rich DNA (except MB3T4G and MB3G4T) are due to the presence of various i-motif-like conformers for a particular template, which is the reason for the occurrence of distinguished absorption and fluorescence emission of Ag-NC on each scaffold, as was observed in Figure 1 and Figure 2 and Table 1.

### 2.2. Influence of pH and Increasing Silver Concentrations on the Growth of Species I, II, III

**Figure 4:**
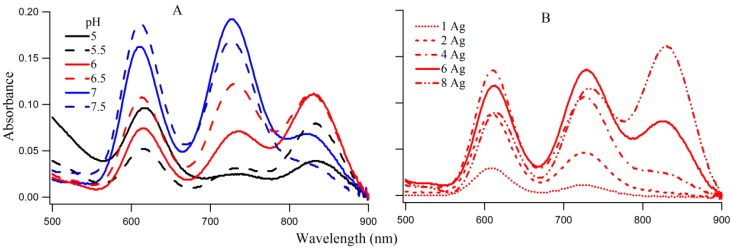
**(A**) Absorption spectra of silver nanoclusters on 3T4G template (15 μM DNA and 90 μM AgNO_3_ and 90 μM NaBH_4_) in 10 mM citrate buffers of pH 5, 5.5, 6, 6.5, 7, and 7.5; (**B**) Influence of increasing [Ag] on the absorption spectra of 3T4G templated Ag-NCs in buffer of pH 6.5. Species I, II, III correspond to the absorption bands at ~620, 730, 830 nm.

The coexistence of species I, II, and III on 3T4G makes this DNA template an ideal candidate to study the interconversion between the three species. The absorption spectra of 3T4G NC (with 15 μM DNA and 90 μM AgNO_3_ and NaBH_4_) with increasing pH from 5 to 7.5, are presented in Figure 4A. With increase in pH from 5 to 5.5, absorbance of species I decreased with increase in II and III (more than II). At pH 6 and 6.5, species III has the highest absorbance, which decreases at higher pH (7 and 7.5), with a simultaneous growth in the absorbances of I and II. The increase in the growth of species III with increase in pH until pH 6.5 suggests that species III forms maximally at low H^+^. This indicates that a balance between H^+^ and Ag^+^ is needed to form species III. Circular dichroism studies indicated that the template Ag-NC conjugated 3T4G forms a classic i-motif at pH 6.5 (Figure 3B), which drives the formation of species III. This agrees very well with the findings of Day *et al.* [27], where Ag^+^ ions induced i-motif folding has been observed at neutral and basic pH conditions. This is explained in detail using CD spectroscopic measurements as given below.

Figure 4B provides the absorption spectra of Ag-NC grown on 3T4G using different concentrations of Ag^+^. It is observed that with increase in [Ag^+^] from 15 (DNA:Ag = 1:1) to 120 μM (DNA:Ag = 1:8), there is an increase in growth of species I, II, and III. Species III appears to start forming at Ag^+^ concentration of 60 μM and at 120 μM it has the highest absorbance. This is indicative of the association of a larger Ag cluster with species III. We studied the aging of the nanocluster species for the 1DNA: 6Ag samples of 3T4G, for five days which is shown in Figure S3. We observed that absorbance of species II and III decreased over time with a simultaneous increase in species I. This is due to the conversion of the templates from actual i-motif to mostly i-motif-like topologies with time. The freshly prepared 3T4G enclosed Ag-NC solution has mixture of all conformers of i-motif (i-motif-like) including the classical i-motif (as discussed in the previous section on CD spectroscopy), which slowly converts to i-motif-like forms, thereby increasing the concentrations of Species I. It is pertinent to mention that species II is previously shown to be made up of 10 Ag atoms [20].

**Figure 5:**
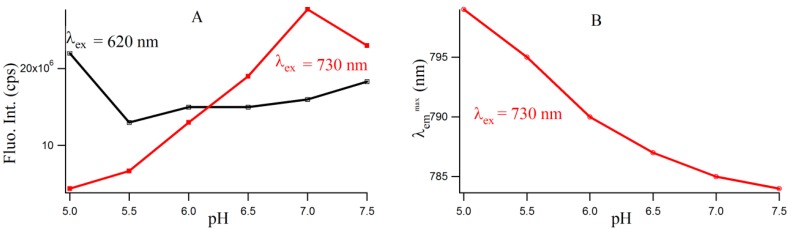
(**A**) Variation of fluorescence intensities with increasing pH for 3T4G template silver nanoclusters. The black (with open squares) and red (with solid squares) lines denote species I (λ_ex_ = 620 nm/λ_em_ =700 nm) and II (λ_ex_ = 730 nm/λ_em_ = 800 nm), respectively; (**B**) Variation of $\lambda_{\text{em}}^{\text{max}}$ of species II with increasing pH (red line with circles).

**Figure 6:**
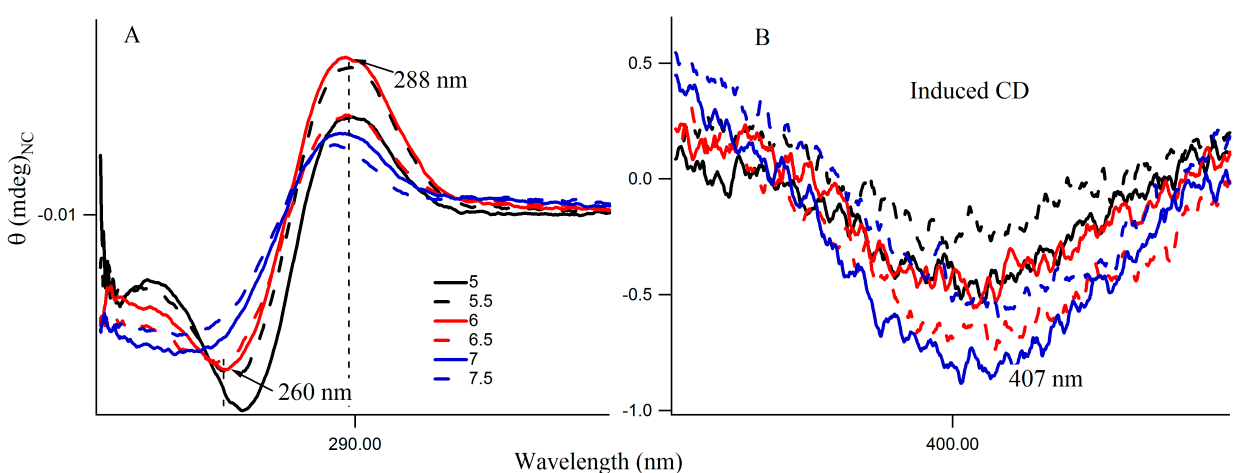
(**A**,**B**) Circular Dichroism (CD) spectra of 15 µM 3T4G template with 90 μM AgNO_3_ with 90 μM NaBH_4_ in citrate buffer at different pH. The region between 350 and 450 nm is enlarged in Figure (**B**).

Figure S4 provides the fluorescence emission spectra of 3T4G template Ag-NCs in different pH solution. Fluorescence intensity of species II increases until pH 7, which is shown by the red profile in Figure 5A. For species I, there is a little increase in fluorescence intensity with increase in pH from 5.5 to 7.5. However, there is a sharp blue shift is the $\lambda_{\text{em}}^{\text{max}}$ of species II (as is shown Figure 5B) from 799 (pH 5) to 787 (pH 6.5), which gradually levels off at higher pH. This could be either due to the dipolar relaxation process, indicating the less polar surrounding matrix around the excited NC fluorophore at higher pH and/or due to the change in the structure of the microenvironment. The former is discussed in the next section. To clarify the possible reason, we looked at the secondary structure of the nanocluster scaffold at different pH. Figure 6A shows the CD spectra of Ag nanocluster conjugated 3T4G template at pH 5–7.5, where signatures of i-motif-like structures are evident in all samples. However at pH 6 and 6.5, the classic profiles of i-motif (negative and positive bands at 260 and 288 nm) could be observed, which supports our findings from Figure 4A,B that species III needs an accurate i-motif template, which only takes place at the correct combination of Ag^+^ and H^+^ ion concentrations. Figure 6B magnified the Figure 6A spectra between 350 and 450 nm, where nanocluster induced asymmetric nature of the template can be observed. At higher pH (7, 7.5), where the i-motif features become weaker (according to Figure 6A), the absorbance of species III decreased, which was shown in Figure 4A. Hence species I and II are grown in the loosely folded i-motif-like structures which mainly exist at neutral to slightly basic pH.

Fluorescence emission spectra of species I and II with increasing concentrations of Ag^+^, when excited at 620 (I) and 730 (II) nm, respectively, are shown in Figure S5, where the changes are consistent with the changes in absorbance (Figure 4B). Although no significant change in the $\lambda_{\text{em}}^{\text{max}}$ of species I is observed, there is a 5 nm red shift in the $\lambda_{\text{em}}^{\text{max}}$ of species II for the 1:8 ([DNA]:[Ag]) 3T4G nanoclusters. There are two possible reasons for this: i. An appreciable change in the microenvironment of species II at higher Ag^+^ concentrations, ii. The interference of the fluorescence of species III, due to its higher absorption at higher Ag^+^. In the earlier section we have observed that the growth of species III is associated with the correct folding of the template in an i-motif manner at higher Ag^+^ and lower H^+^ concentrations. The red shift of the $\lambda_{\text{em}}^{\text{max}}$ of species II at higher Ag^+^ is thus significantly associated with both the change in microenvironment of the Ag-NCs and interference from species III. The influence of different salt (NaNO_3_) concentrations on the formation of the 3T4G enclosed Ag-NC was studied, using absorption and CD spectroscopy, which are shown in Figure S6. With increase in salt from 60 to 100 mM, although no change in the secondary structure of the template was observed, there was a significant decrease in the absorbance of species I, II, and III. With further increase in salt to 200 mM, an appreciable increase in the helicity of 3T4G scaffold was observed, but the absorbances were further reduced. It is noteworthy that there was no change in the absorbance of the Ag nanoparticle at ~420 nm at the increased salt conditions. These results suggest that higher concentrations of Na^+^ ions prevent the formation of Ag-NC on the template which reduces the overall productions of species I, II, and III.

### 2.3. Influence of the Polarity of the Microenvironments on Species I, II, III

**Figure 7:**
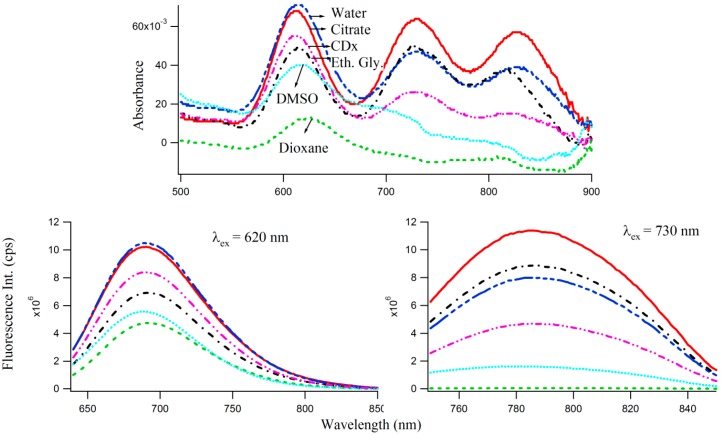
Influence of solvent polarities on the absorption (**top**) and fluorescence (**bottom**) spectra of 7.5 μM3T4G templated Ag-NCs (with 45 μM AgNO_3_ and 45 μM NaBH_4_). Descriptions of the lines are given in the top figure (absorption spectra) which are same in bottom (emission spectra of Species I (**left**) and Species II (**right**)).

One of the motivations of undertaking the present study is to inquire whether one can observe manifestations of dipolar relaxation processes [22,24,25,28] around the excited DNA enclosed Ag-NC molecules when incorporated in the matrices of varied polarities. Solvent dipolar reorientation, which responds strongly to variations in the fluorophore environments [24,25], can be an important relaxation mechanism for the excited S_1_ state of the Ag-NC. A convenient way to analyze dipolar relaxation process by steady state fluorescence spectroscopy is to mix the NC in a confined environment such as cyclodextrin. The use of cyclodextrin (both naturally derived, and chemically modified varieties) as “nano vehicles” for therapeutic drugs [29,30,31], has become a common method in recent years due to their capabilities of enhancing solubility of hydrophobic drugs, their dissolution rate, and increased membrane permeability. With a hydrophilic outer surface and comparatively hydrophobic central cavity, cyclodextrins can form water soluble inclusion complexes with a variety of molecules. In this study, we have used methyl-beta-cyclodextrin (CDx) [29] as well as polar protic (e.g., water, buffer, ethylene glycol) and aprotic (dioxane, DMSO) solvents to study the effect of microenvironments of varied polarities, on the optical properties of 3T4G enclosed Ag-NCs, which are displayed by the absorption and fluorescence spectra in Figure 7. Interestingly, the absorbance maxima (Figure 7 top) of species I blue shifts by 3 nm in CDx compared to that in water/buffer. The CD spectrum of 3T4G templated Ag-NC in the presence of CDx media (not shown) is similar to that in buffer, suggesting that the secondary structure of the DNA template is not altered by CDx [31]. Fluorescence anisotropy (*r*) [22,31] was determined using Equation (1). “*r*” of species II was found to be 0.24 and 0.26 in buffer and CDx matrix, respectively. “*r*” is a measure of the rigidity of the surrounding environment [22]. A low value of “*r*” is indicative of considerable motional freedom of the fluorophore, where “*r*” = 0.4 is the theoretical maximum limit [22,25,31]. The “*r*” of 0.24 suggests that Ag-NC are motionally constrained inside the DNA template, and this restriction increases in CDx environment. Polar aprotic solvent DMSO only allowed the formation of species I of 3T4G Ag-NC. Dioxane destabilized the DNA template (as was evident from CD spectra, not shown) and hence the cluster formation did not take place. However, there is a 3 nm red shift in the emission maxima of species I and II in protic solvent ethylene glycol (relative polarity 0.794) [25] with respect to buffer, which implies that 3T4G templated Ag-NC can appreciably probe the environmental variations. Hence, the shifts in $\lambda_{\text{em}}^{\text{max}}$ of 3T4G enclosed Ag-NCs observed in Figure 5B can also be attributed to a difference of the dipole moments between ground (S_0_) and excited (S_1_) states, resulting in noticeable solvent dipolar relaxation around the chromophore [25]. We studied the stabilities of the species I, II, and III in all the solvents for three consecutive days using absorption and fluorescence parameters. Figure S7 shows that the intensities of species I increases at the cost of II and III. This increase is more in ethylene glycol environment and may be attributed to the viscous nature of the solvent. This opens the door to the use of viscous solvents in tuning and growing of an intended NC species.

### 2.4. Size Exclusion Chromatographic (SEC) Studies

**Figure 8:**
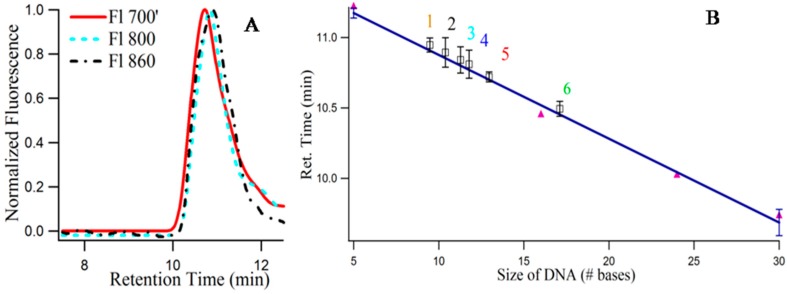
(**A**) Size exclusion chromatogram of the three fluorescent Ag nanocluster species of template 3T4G: Red (solid), cyan (- - -), and black (-.-.-) lines correspond to λ_ex_ = 620/λ_em_ = 700 nm (I), (λ_ex_ = 730/λ_em_ = 800 nm (II) and λ_ex_ = 830/λ_em_ = 860 nm (III); (**B**) Calibration curve using the single-stranded thymine oligonucleotides dT_5_, dT_16_, dT_24_, and dT_30_ (purple solid circles) as the size standards to relate the column retention times to the log of molecular mass of the silver nanoclusters of 1. MB3T4G (λ_ex_ = 540/λ_em_ = 620 nm); 2, 3, and 5 correspond to Species III, Species II, Species I of 3T4G in buffer; 6. Species I of 3T4G in methyl-beta-cyclodextrin; 4 denotes the retention time of the 3T4G free DNA (using absorbance value at 260 nm). Each data point is average of three independent measurements and the bars indicate the standard deviation.

Although, the importance of the secondary structures (folding) of the single stranded DNA templates on the growth of the NIR emitting Ag-NCs were emphasized by pH and Ag^+^ concentration dependent studies using absorbance, fluorescence, and circular dichroism spectroscopy, the molecularity of the templates remained unknown. SEC provides an easy way to understand the folded nature along with the number of templates associated with the species I, II, III [16]. Figure 8A provides the size exclusion chromatogram (using fluorescence) of 3T4G NC species I (λ_ex_ = 620/λ_em_ = 700 nm), II (λ_ex_ = 730/λ_em_ = 800 nm) and III (λ_ex_ = 830/λ_em_ = 860 nm). It is noteworthy that, due to the sensitivity limitation of the detector used for fluorescence measurements in the NIR region, the exact $\lambda_{\text{em}}^{\text{max}}$ of species III are not known. Hence, λ_em_ = 860 nm for detecting species III in the fluorescence detector of SEC (see Materials and Methods), is an approximation. The retention times of species III (10.89 min) > II (10.84 min) > I (10.72 min) prove that species III has the most compact structure, which agrees very well with our findings from CD spectroscopic measurements which showed that species III required a fully folded, classical i-motif to form. Single stranded thymine (T) oligonucleotides of different sizes (5, 16, 24, 30 bases long) were used to draw a standard curve, with the assumption that thymine oligonucleotides do not undergo folding [16]. Figure 8B presents the best fit line of the plot between retention time and log of molecular mass of “T” DNAs. Using slope and intercept of the standard curve, the “observed molecular mass” of the unconjugated 3T4G was calculated to be 2630 (designated by “4” in Figure 8B), which is around half of its actual molecular mass (4668), confirming the folded nature of the free unimolecular template. CD spectra of the unconjugated DNA at pH 6.5 (Figure 3A) showed weak i-motif folding, which is confirmed by SEC. Similarly, the “observed molecular mass” of MB3T4G templated Ag-NC (using fluorescence with λ_ex_ = 540 nm) was found to be 2209, compared to the actual mass of 7138 g/mole, which is consistent with the hairpin like (Scheme 1B) folded structure for MB3T4G. The mass of the species I, II, III (Figure 8A) were calculated to be 2948, 2531, 2366 (shown by “5”, “3”, “2” in Figure 8B). The molecular mass of CDx bound species I of 3T4G-NC (“6” in Figure 8B) was found to be 3936, which proves that one cyclodextrin molecule conjugates with the Ag-NC species (the average molecular mass of CDx is ~1310). Species I and II must be associated with i-motif-like topologies. SEC measurements confirmed the uni-intramolecular folding nature of the templates and supported the findings from CD spectroscopic studies.

## 3. Materials and Methods

The clusters are synthesized using oligonucleotides (Integrated DNA Technologies), which were purified by standard desalting by the manufacturer. Seventeen oligonucleotides were used for the study as is shown in Scheme 1. All solvents are obtained from Sigma and of spectrograde quality and used without further purification. Deionized water is autoclaved. Concentrations of the oligonucleotides were determined by absorbance using molar absorptivities based on the nearest-neighbor approximation. Silver clusters were synthesized by combining 15 µM DNA (the solution was kept in boiling water for 5 min to break all intramolecular interactions) and 90 µM Ag^+^ solutions (except mentioned otherwise) in a 10 mM citrate buffer (Sigma-Aldrich, St. Louis, MO, USA) at pH = 6.5 [16,17] Then, an aqueous solution of BH_4_^−^ was added to give a final concentration of 6 BH_4_ /oligonucleotide, and the resulting solution was vigorously shaken for 1 min. The samples reacted overnight in the dark at 4 °C. Methyl-beta-cyclodextrin (CDx, Sigma) solution was made by dissolving the solute in citrate buffer. To explore the influence of solvents (with varied dielectric constants) and a confined matrix CDx, on the formation and behavior of the Ag-NCs, 50:50 (*v*/*v*) mixtures of 3T4G scaffolded Ag-NCs in citrate buffer were prepared in the polar protic, aprotic solvents, and CDx solution, which were then incubated at 4 °C for overnight, following the same steps as mentioned before. For salt dependent studies, NaNO_3_ was used.

Steady state absorption spectra were recorded with Shimadzu UV 2550 spectrophotometer (Kyoto, Japan). Steady state fluorescence measurements were carried out with Fluoromax-4 (Horiba Jobin Yvon, JobinYvon, NJ, USA) spectrofluorometer, equipped with polarizers and a Peltier temperature controlled cell. Excitation and emission slit widths were 3/3 nm. All reported luminescence spectra were corrected for the spectral response of the detector.

Steady state fluorescence anisotropy (*r* [22,25]) values were calculated using the expression

(1) $$r=\frac{I_{VV}-GI_{VH}}{I_{VV}+2GI_{VH}}$$

where *I_VV_* and *I_VH_* are the vertically and horizontally polarized components of the Ag-NC emission intensity after excitation by vertically polarized light at the respective wavelength. For 3T4G “*r*” is measured at λ_ex_ = 620 nm/λ_em_ = 700 nm and λ_ex_ = 730 nm/λ_em_ = 800 nm with slit widths 5/5 nm in buffer and CDx environments.

Fluorescence lifetimes (τ) were measured by time correlated single photon counting using an OB920 spectrometer (Edinburgh Instruments Ltd., Livingston, UK) in conjunction with a pulsed LED (PicoQuant, Berlin, Germany) emitting at 460 nm or a pulsed laser diode (PicoQuant) emitting at 659 nm as excitation light source. In terms of rate constants, τ is measured following the relation below, where *k*_r_ and *k*_nr_ are the radiative and non-radiative rate constants [22,24,25]:

(2) $$\tau =\;\frac{1}{k_{r}+\;k_{nr}}$$

Circular dichroism (CD) spectra were acquired with a J-810 spectropolarimeter (Jasco, MD, USA). The scan rate was 100 nm/min, and three consecutive spectra were averaged to produce the final spectrum. All spectral measurements were performed at 25 °C in 0.02 cm path length Hellma cell. The highest concentration of DNA for fluorescence and circular dichroism experiments were kept at 10–15 µM to avoid aggregation, scattering, and artifacts.

Size exclusion chromatography (SEC) used a 300 × 7.8 mm i.d. column (BioSep, 3000, Phenomenex, Torrance, CA, USA) on an HPLC system (SCL 10A VP, Shimadzu, Kyoto, Japan) using a 10 mM citrate with 100 mM NaClO4 buffer at pH 6.4 to minimize matrix adsorption [16]. For the thymine oligonucleotides dT5, dT16, dT24, dT30, the averages of the retention times, and the corresponding molecular masses were fitted linearly, from which the folded nature of the 5′-d(C3X)4-3′ DNA oligonucleotides were determined, assuming that there were no secondary interactions within the thymine oligonucleotides. Here, the molar masses of the thymine oligonucleotides were used as standards for drawing a calibration plot to obtain the molecular masses of the free and bound DNAs for stoichiometric purposes. Absorption and fluorescence measurements of the species were made using the SPD-10AVi and RF-10AXL Shimadzu detectors (with measuring wavelength range 200–900 nm, and spectral bandwidth 15 nm both in the excitation and emission sides and accuracy ± 2 nm), respectively. The time difference between the two detectors was adjusted [16,17]. The injection volume was 20 μL. Three or more chromatographs were acquired to determine an average retention time.

## 4. Conclusions

The structural polymorphism shown by i-motif DNA is regulated by number of cytosine bases in the stem, the nature of base and length in the loop and the polarity of the surroundings. This is manifested by i-motif-like circular dichroism spectra and distinguished features in the absorption and fluorescence emission profiles of the oligonucleotide enclosed silver nanoclusters. We observed that a single base mutation in the single stranded DNA scaffolds dramatically altered the absorption, steady state, and time resolved fluorescence emission properties of the enclosed Ag-NCs. Double exponential fluorescence decay characteristics are indicative of heterogeneity in the microenvironments of the fluorophore Ag-NC. Addition of complementary sequences at both the ends of the template (making a hairpin structure) changed the cluster’s fluorescence emission and decay properties drastically. [DNA]:[Ag] stiochiometric and pH dependent studies reveal that the right combination of H^+^ and Ag^+^ dictate the formation of the NIR absorbing species III, which only forms on an intramolecularly folded classical i-motif template of 3T4G. Species I and II are associated with i-motif-like templates. This systematic, detailed, and exploratory study focuses attention on novel features of the DNA templates of Ag-NC, which strongly suggest their potential usefulness as probes for studying complex systems. Further investigations are being contemplated in order to obtain more detailed insights regarding the emission properties of the longer NIR species.

## Figures and Tables

**Table 1:** Absorption maxima ($\lambda_{\text{abs}}^{\text{max}}$) along with absorbance (a.u) and fluorescence emission maxima ($\lambda_{\text{em}}^{\text{max}}$, corrected) along with the intensities (cps) of the silver nanocluster species formed on the 15 μM single stranded DNA templates ([DNA]:[Ag] = 1:6) with the repeated base sequence 5′-C_3_X_i_C_3_X_ii_C_3_X_iii_C_3_X_iv_-3′ where i, ii, iii, iv correspond to T/G/C deoxynucleobases (with default base X = A) in 10 mM citrate buffer of pH 6.5.

Templates	$\lambda_{\text{abs}}^{\text{max}}$ (nm)	$\lambda_{\text{abs}}^{\text{max}}$ (nm)	$\lambda_{\text{abs}}^{\text{max}}$ (nm)	$\lambda_{\text{em}}^{\text{max}}$ (nm) (λ_ex_ = 620)	$\lambda_{\text{em}}^{\text{max}}$ (nm) (λ_ex_ = 730)	$\lambda_{\text{em}}^{\text{max}}$ (nm) (λ_ex_ = 460)	$\lambda_{\text{em}}^{\text{max}}$ (nm) (λ_ex_ = 540)
1T	652 (0.10)	725 (0.05)		704 (1.50 × 10^7^)	781 (9.27 × 10^6^)		
1T4G	656 (0.10)	730 (0.10)		698 (1.70 × 10^7^)	787 (1.88 × 10^7^)		
2T	640 (0.05)	714 (0.03)		698 (9.90 × 10^6^)			
2T4G	651 (0.07)	723 (0.08)		696 (1.20 × 10^7^)	782 (1.38 × 10^7^)		
2G4T	550 (0.04)	626 (0.04)		697 (5.55 × 10^6^)	775 (1.08 × 10^6^)		
3T4G	610 (0.10)	727 (0.15)	828 (0.16)	688 (9.10 × 10^6^)	784 (2.33 × 10^7^)		
3T	630 (0.04)	730 (0.25)		697 (3.05 × 10^6^)	783 (3.27 × 10^7^)		
3T4T		723 (0.23)		698 (1.44 × 10^6^)	780 (3.88 × 10^7^)		
3G	625 (0.04)	716 (0.31)		698 (3.69 × 10^6^)	773 (4.4 × 10^7^)		
3G4T	622 (0.03)	716 (0.29)		698 (3.15 × 10^6^)	772 (4.05 × 10^7^)		
4T	638 (0.05)	725 (0.20)		697 (5.96 × 10^6^)	779 (3.04 × 10^7^)		
4G	620 (0.10)	737 (0.18)	832 (0.07)	697 (2.05 × 10^7^)	794 (2.67 × 10^7^)		
C_3_TIM		520 (0.07)					640 (2.77 × 10^6^)
C_16_	463 (0.15)						657 (1.39 × 10^6^)
3C		728 (0.03)		705 (9.98 × 10^5^)			614 (3.57 × 10^5^)
MB3T4G	460 (0.139)	542 (0.133)		690 (3.6 × 10^5^)	793 (1.31 × 10^5^)	563 (2.07 × 10^7^)	619 (1.81 × 10^7^)
MB3G4T	464 (0.33)			710 (4.36 × 10^5^)	771 (2.46 × 10^5^)	596 (3.45 × 10^7^)	

**Table 2:** Fluorescence decay parameters of the silver nanocluster species formed on the 15 μM single stranded DNA templates ([DNA]:[Ag] = 1:6) with the repeated base sequence 5′-C_3_X_i_C_3_X_ii_C_3_X_iii_C_3_X_iv_-3′ where i, ii, iii, iv correspond to T/G/C deoxynucleobases (with default base X = A) in 10 mM citrate buffer of pH 6.5.

Templates	λ_ex_ = 659 nm λ_em_ = 700 nm			λ_ex_ = 460 nm λ_em_ = 535 nm			λ_ex_ = 496 nm λ_em_ = 600 (620) ^ nm		
	($\bar{\tau }$)	τ_1_	τ_2_ (ns)	($\bar{\tau }$)	τ_1_	τ_2_ (ns)	($\bar{\tau }$)	τ_1_	τ_2_ (ns)
3T	2.4	1.2 (28%)	2.8 (72%)						
4G	2.4	1.2 (22%)	2.7 (78%)						
3T4G in buffer	2.4	7.9 (3%)	2.3 (97%)						
3T4G in CDx ^a^	2.3	6.6 (3%)	2.2 (97%)						
MB3T4G ^				1.7	2.8 (41%)	1.0 (59%)	4.6	1.7 (18%)	5.4 (82%)
C_3_Tim							4	0.6 (21%)	4.8 (79%)
C_16_							4	1.4 (37%)	5.4 (63%)

^a^ 50% Methyl-beta-cyclodextrin. ^ For MB3T4G, the λ_em_ was 620 nm.

## Supplementary Materials

Supplementary materials can be accessed at: http://www.mdpi.com/1420-3049/21/2/216/s1.
