# Supplementary material for: DNA Scaffolded Silver Clusters: A Critical Study
Source: Molecules. 2016 Feb 17;21(2):216. doi: 10.3390/molecules21020216 (PMC6274378; doi:10.3390/molecules21020216)
Supplement: Supplementary file 1 [file molecules-21-00216-s001.pdf]

Supplementary Materials: DNA Scaffolded Silver Clusters: A Critical Study

Bidisha Sengupta, Christa Corley, Keith Cobb, Anthony Saracino, Steffen Jockusch

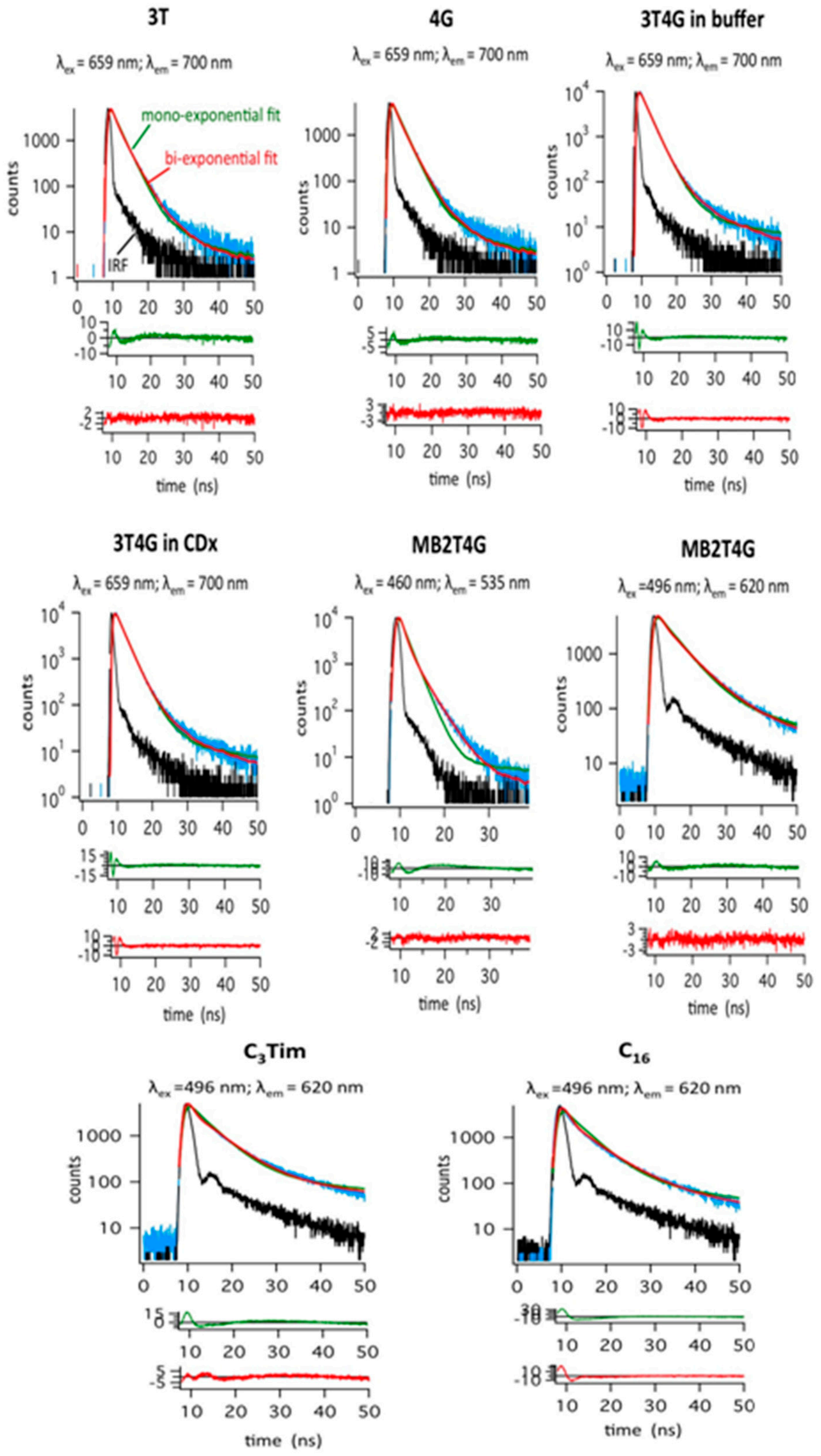

Figure S1. Fluorescence decay profiles of Ag-NC on different DNA templates.

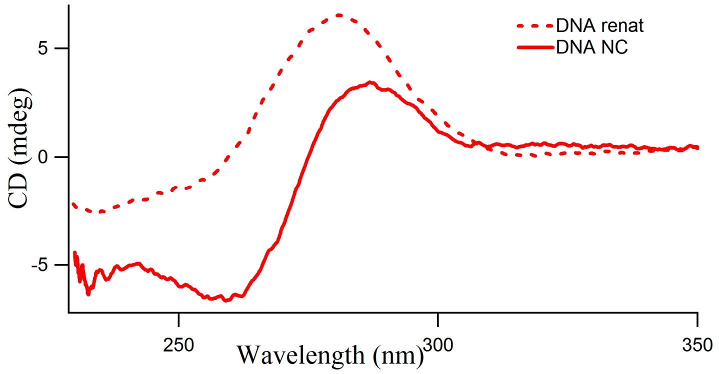

Figure S2. CD spectra of renatured 3T4G template in unconjugated (dashed) and Ag-NC conjugated (solid) states.

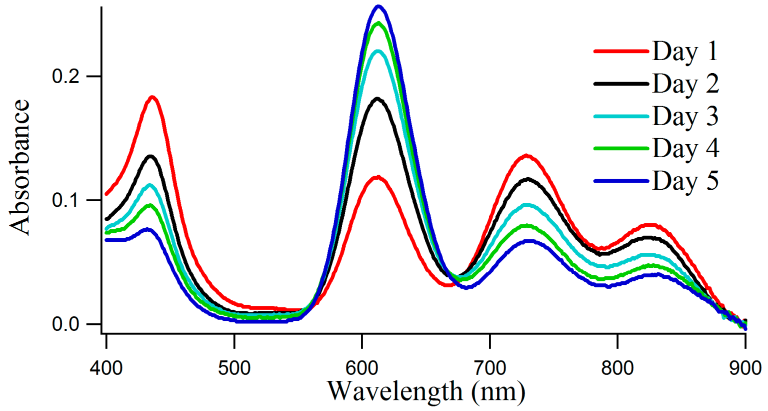

Figure S3. Aging of the 3T4G Ag-NCs for [DNA]:[Ag] = 1:6, for 5 days.

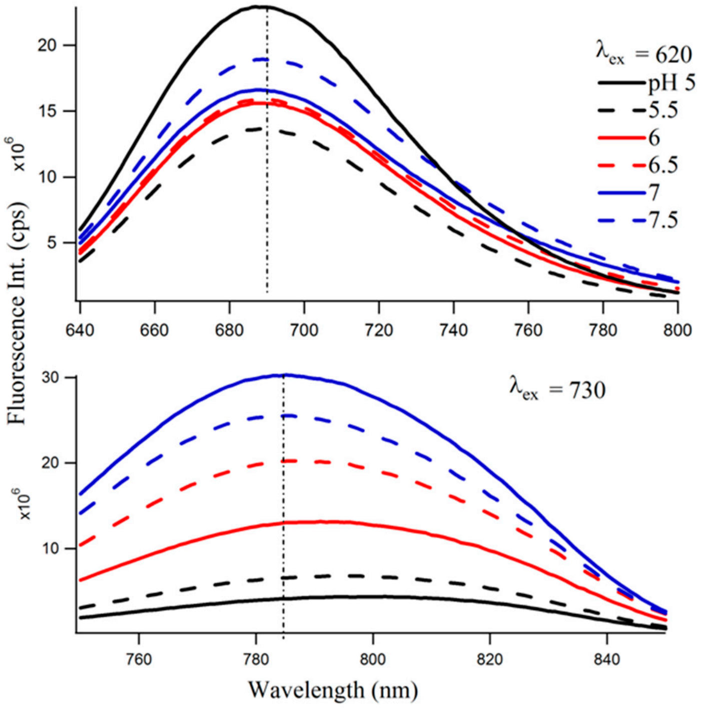

Figure S4. Fluorescence emission spectra of Ag-nanoclusters on 3T4G template in 10 mM citrate buffer of various pH.

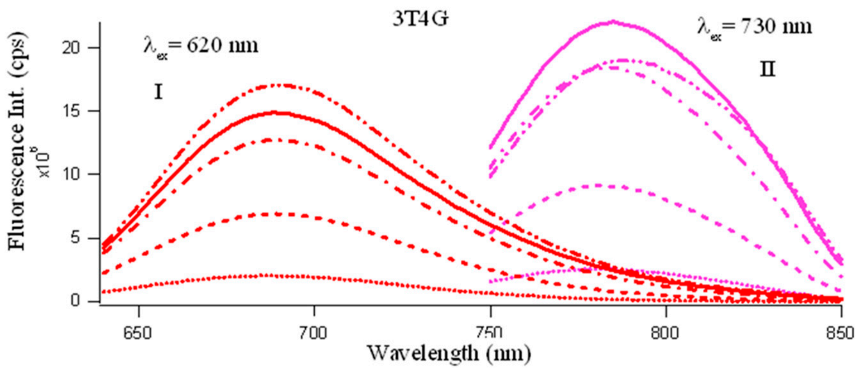

Figure S5. Influence of increasing silver concentrations on the fluorescence emission properties of the silver nanoclusters on 3T4G DNA templates in 10 mM citrate buffer of pH 6.5. [3T4G]:[Ag] is 1:1 (……), 1:2 (- - -), 1:4 (-.-.-), 1:6 (—), 1:8 (-..-).

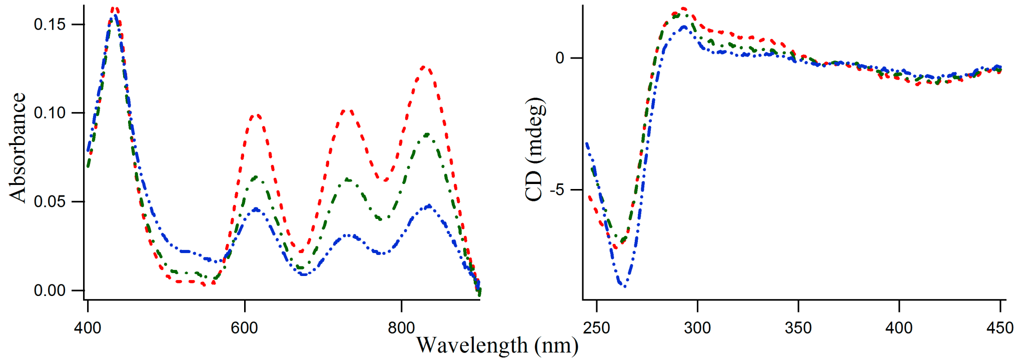

Figure S6. Absorption (**left**) and circular dichroism (**right**) spectra of 3T4G templated Ag-NC in citrate buffer of pH 6.5 with $NaNO_3$ salt concentrations of 60 (red - - -), 100 (green -.-.-.) and 200 (blue -..-..-..-..) mM.

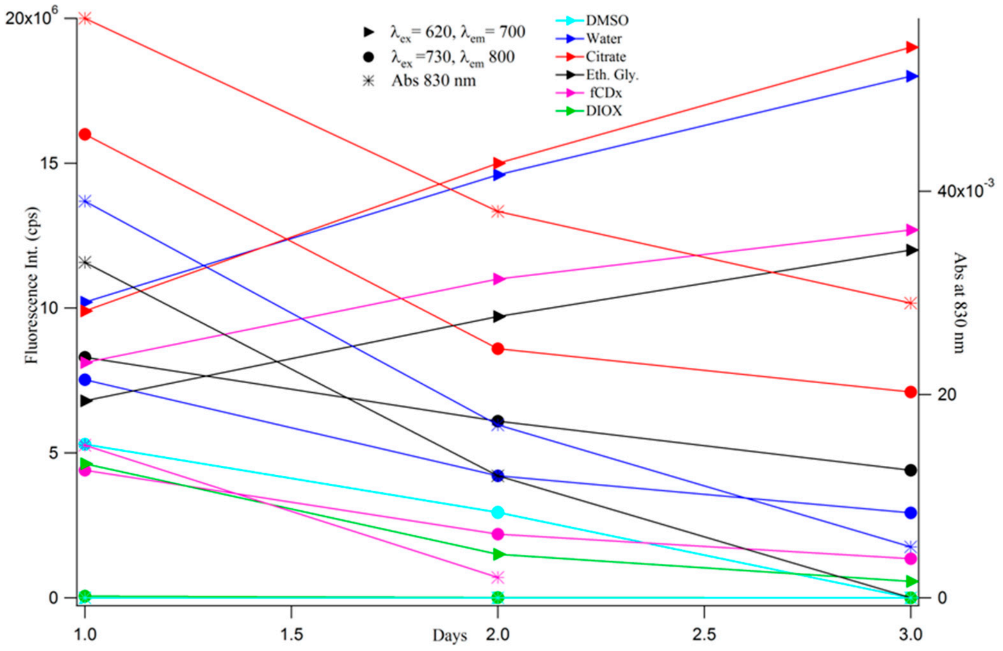

Figure S7. The variation of the fluorescence emission intensities and absorbance of 3T4G scaffolded Ag-NC species I (λ_{ex} = 620), II (λ_{ex} = 730) and III ((λ_{abs} = 830) with time (days) in solvents of different polarities (7.5 μM 3T4G enclosed Ag-NCs with 45 μM $AgNO_3$, 45 μM $NaBH_4$).
